# The impact of food insecurity on mental health among older adults residing in low- and middle-income countries: A systematic review

**DOI:** 10.1371/journal.pone.0301046

**Published:** 2024-03-26

**Authors:** Cornelius Osei-Owusu, Satveer Dhillon, Isaac Luginaah

**Affiliations:** 1 Schulich School of Medicine and Dentistry, Western University, London, Ontario, Canada; 2 Department of Geography and Environment, Western University, London, Ontario, Canada; University of Helsinki, FINLAND

## Abstract

Over the past few years, food insecurity has been increasing globally due to the COVID-19 pandemic, climate change, economic downturns and conflict and a number of other intersecting factors. Older adults residing in low- and middle-income countries are more vulnerable to food insecurity. While the impacts of food insecurity on physical health outcomes have been thoroughly researched, the effect on mental health outcomes remains under-researched, especially among older adults. Hence, this systematic review aims to investigate existing literature to assess how food insecurity impacts the mental health of older persons residing in LMICs. A systematic search of six databases and Google for studies was conducted. The search was limited to studies written in English and published between 2000 to the present. We identified 725 studies, out of which 40 studies were selected for a full-text review and 12 studies were included for a final analysis. The significant finding in all the included studies was that food insecurity is associated with the worsening mental health of older adults. We also found a complex interplay of factors such as gender, age, rural/urban and health conditions associated with the aggravation of several mental health outcomes. The findings of this study illuminate the need for improved food programs to improve food security and, consequently, mental health among older adults.

## Introduction

Food insecurity is defined as the limited or uncertain availability of sufficient, safe, and nutritious food due to limited physical and economic access [1, 2]. Food insecurity has become an increasing public health concern, exacerbated by the COVID-19 pandemic, economic changes, climate change and impacts on the global food chain [3–5]. According to the World Food Programme, more than 345 million people are facing high levels of food insecurity in 2023, a number that is double the number in 2020 [6]. While this is a global issue, low and middle-income countries (LMICs), defined by the World Bank as countries with a Gross National Income per capita below $13,205, are most vulnerable to food insecurity [7].

Food insecurity is caused by a number of complex factors that often intersect. Within the context of LMICs, the COVID-19 pandemic contributed to food insecurity. For example, in India COVID-19 related public health measures impacted the transportation of food products, slowed down the food processing industry due to labor shortages and social distancing guidelines, and the closure of food markets and trade restrictions resulted in reduced food availability and price volatility [8–10]. In other regions, the impact was similar. For instance, South Africa had high levels of food insecurity even before the COVID-19 pandemic, and due to the COVID-19 pandemic, food insecurity worsened [11, 12].

LMICs, compared to higher-income countries, will be disproportionately impacted by climate change, and climate change is a determinant of food insecurity through a number of multifactorial mechanisms [13, 14]. Climate change directly impacts food production through changing agroecological conditions, affecting people’s livelihoods and the stability of food supplies [15]. For instance, slow-onset climatic events in Latin America and the Caribbean, such as soil erosion and decline in farmland yield, negatively impact agricultural production, increasing food insecurity [16, 17].

Several other factors, such as economic downturns and conflict compounded with climate change and the COVID-19 pandemic, further exacerbate food [18, 19]. The current Russian military aggression against Ukraine, an exporter of many food supplies, has disrupted food chains worldwide [20]. Several LMICs import supplies, such as wheat, from Ukraine, and due to the invasion, there has been a shortage of around 30 million tons of grain in Africa, along with an increase in cost [21]. Rising food costs due to the economic crisis further weaken the ability of households to access adequate food [22].

Further, LMICs are undergoing a rapid demographic transition due to population ageing [23]. Estimates state that by 2050, 80% of older adults will reside in LMICs [24]. Older adults residing in LMICs often have no social safety nets, no fixed income, inadequate access to health infrastructure, and are not priorities for the government [25–27]. As a result, older adults are more vulnerable to food insecurity [28, 29].

Food insecurity is linked to several physical health outcomes, including stunting [30, 31], chronic disease [32], and obesity [33]. While the impact of food insecurity on physical health outcomes has been researched in several contexts, the impact food insecurity has on mental health remains under-researched, especially among older adults. According to the World Health Organization, mental health is “*the state of mental well-being that enable people to cope with the stresses of life*, *realize their abilities*, *learn well and work well*, *and contribute to their community* [34].” Examples of mental health conditions include anxiety disorders, depression, bipolar disorder, and neurodevelopmental disorders [34]. Mental disorders negatively impact the quality of life of those affected and relate to significant socioeconomic burdens [35, 36]. For older adults, mental health conditions can increase the risk of age-related impairments and mortality [37]. However, mental health remains a neglected policy priority, especially for older adults in LMICs [38]. Despite this, there has been no systematic review performed to study the link between food insecurity and mental health outcomes in LMICs.

The Sustainable Development Goals [SDGs] have focused on food insecurity and mental health. For example, SDG 2.1 focuses on “*By 2030*, *end hunger and ensure access by all people*, *in particular the poor and people in vulnerable situations*, *including infants*, *to safe*, *nutritious and sufficient food all year round* [39].” Moreover, SDG 3.4 aims to “*reduce by one-third premature mortality from non-communicable diseases through prevention and treatment and promote mental health and well-being* [39].” Further, the commitment to “leave no one behind” and SDG 5, which focuses on the right to health “for all at all ages,” illuminates the importance of focusing on older adults [40]. Overall, it is essential to understand the links between food insecurity and mental health among the ageing population, as this would help reach the SDG targets and contribute to policy development.

This systematic review aims to investigate existing literature to assess how food insecurity impacts the mental health of older persons residing in LMICs. Specifically, this review will examine i) the relationship between food insecurity and various mental health outcomes among older persons in LMICs and ii) whether meditating factors place specific sub-groups of older adults at an increased risk of negative mental health outcomes due to food insecurity.

## Methods

### Search strategy

This review was registered with the PROSPERO prospective register of systematic reviews database (ID: CRD42023442481) on July 15th, 2023. A detailed, comprehensive literature search was carried out for published articles from Medline (OVID), Embase (OVID), PsycINFO (OVID), CINAHL, and Scopus, Web of Science between the dates June 28th and July 15th, 2023, using the search terms listed in Table 1. The authors generated search terms with the assistance and support of a Research and Scholarly Communications Librarian from Western University (London, Ontario, Canada). Using Godin et al., a grey literature search was also undertaken using Google Advanced [41]. Search strings were modified for each search engine, using Boolean operators to combine terms.

**Table 1:** Abbreviated search strategy for identifying relevant sources on how food insecurity impacts mental health among older adults residing in LMICs.

Domain	Search Terms
Food Insecurity	"food insecurit*" OR hunger OR "food securit*" OR "food ration*" OR "Famine" OR "Food Deserts" OR "food scarcity" OR "dietary insufficiency" OR "hunger" OR "Food Insecurity" OR "Food Security" OR "Food Supply" OR "Nutritional Status" OR "Malnutrition" OR "Poverty" OR "nutritional risk" OR "food availability" OR "food utilization" OR "food access"
Older Adults	"older adult*" OR "aged" OR "middle aged*" OR "aged over fifty" OR "aged over 50" OR "senior*" OR "retire*" OR "older people" OR "older men" OR "older women" OR "retired" OR "geriatric" OR "50 years and older" OR "gerontology" OR "ageing" OR "frail*" OR "ageing" OR "aging" OR "elder"
Mental Health	"Psychological disorders" OR "mental wellbeing" OR "stress" OR "anxiety" OR "social problems" OR "attention deficits" OR "MH" OR "Mental Illness" OR "Mental Disease" OR "Mental Disorder" OR "Depress*" OR "Anxiety Disorders" OR "Post-Traumatic Stress Disorder" OR "Stress Disorders, Post-Traumatic" OR "Bipolar Disorder" OR "depressive symptoms" OR "mental status" OR "quality of life" OR "mental health" OR "mental wellness"
LMICs	"lower income countr*" OR "developing countr*" OR "lower-middle countr*" OR "third world countr*" OR "middle income countr*"

### Citation management

After completing the peer-reviewed literature search, the results were exported into Covidence. In the title and abstract screening stage and full-text screening, each article was individually screened by C.O. and S.D. based on the inclusion and exclusion criteria. Subsequent conflicts were then resolved through a discussion between the two reviewers. For the grey literature search, sources were derived by manually recording into a Microsoft Excel database and one reviewer (S.D) was responsible for title/abstract screening and full-text screening.

### Inclusion and exclusion criteria

Two reviewers (C.O and S.D) applied pre-determined selection criteria for both peer-reviewed and grey literature (see Table 2).

**Table 2:** Selection criteria for identifying relevant sources on food insecurity impacts mental health among older adults residing in LMICs.

	Inclusion	Exclusion
Time Period	• 2000 –Present • The Millennium Development Goals (MDGs), the precursors to the SDGs, were implemented in 2000 [42]	• Before 2000
Geographical Focus	• LMICs as defined by the World Bank (The World Bank, 2022).	• High income countries
Age	• 50 years or age • If studies include all ages, they can be included as long as they do a separate analysis on older adults	• Under 50
Outcomes	• Food insecurity & mental health related	• Studies that focused solely on cognitive development or diseases that affect mental capabilities (i.e., dementia) were excluded • Studies that focused on poverty without connecting it to food insecurity and mental health

### Data extraction

Short-form data were extracted from each study, including the name of authors, publication year, country, World Bank classification, geographic scale, sample size and demographics. Other data that was extracted included: the objective of the study, the methodological approach used, the impact of food insecurity on mental health, key findings, implications for research, limitations of the study and the characteristics of the study that impacted the quality.

### Quality assessment

The quality of each study was evaluated using the National Heart, Lung and Blood Institute (NIH) Study and CASP Quality Assessment Tools. The NHLBI was used as it can be used for a number of various study designs. The assessment criteria for the studies included: the clarity of the objective of the research, eligibility criteria, population of the study, sample size, outcomes that were analyzed and the nature of the statistical analyses that were performed.

## Results

**Fig 1:**
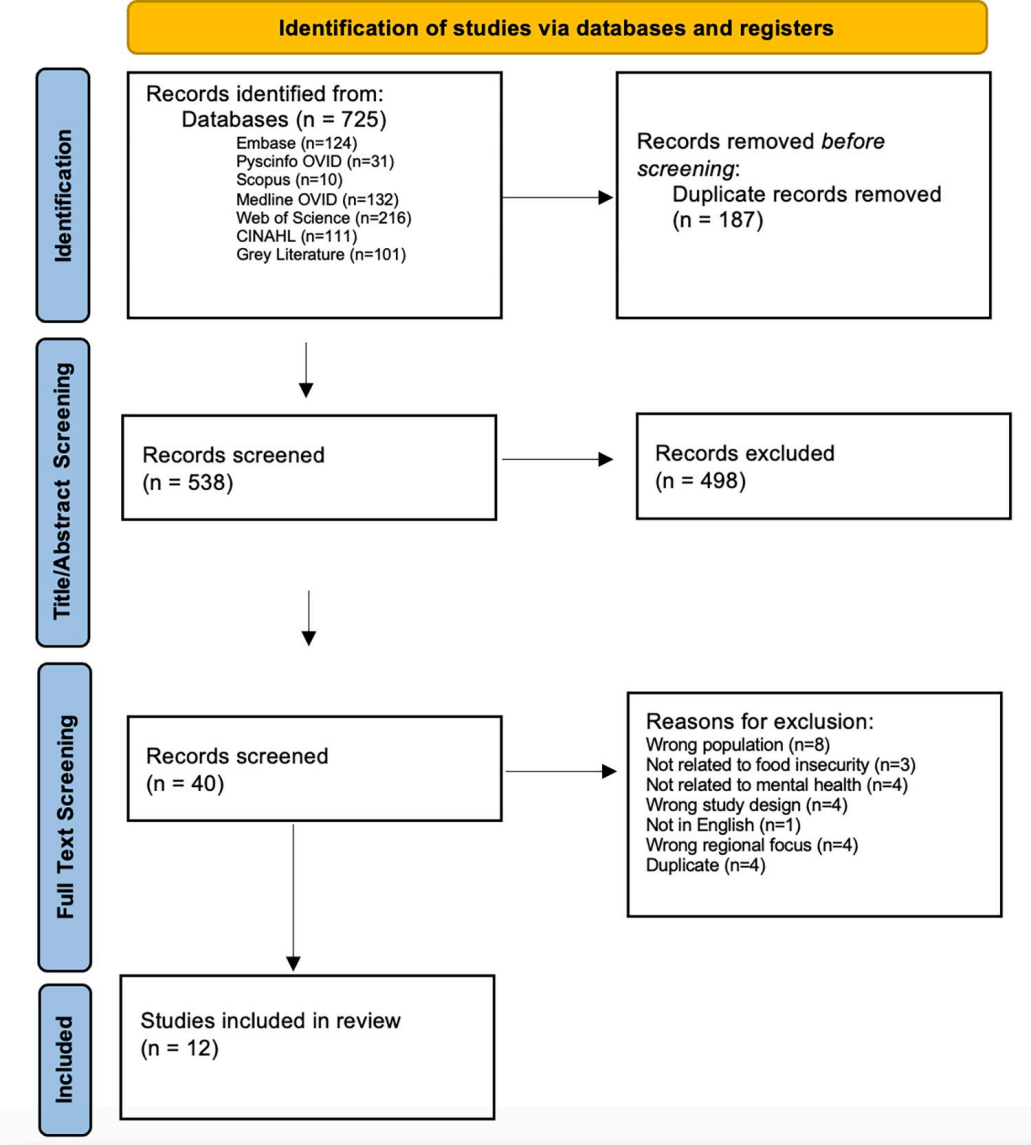
PRISMA chart.

The search yielded 624 articles from 6 databases (EMBASE, PsycINFO OVID, Scopus, Medline OVID, Web of Science and CINAHL) and 101 articles from grey literature (Google searches). 187 duplicates were removed by Covidence before screening. 538 records were screened during the title/abstract screening by two reviewers (C.O. & S.D.), where 498 records were excluded. During the full-text screening, 40 records were screened, and 27 were excluded for various reasons using our inclusion/exclusion criteria. Overall, after completing the quality assessment, 12 studies were included in this review. View Fig 1 for the PRISMA Chart.

### Key attributes of included studies

#### Temporal distribution of articles

The year of publication for the included studies ranged from 2009 to 2023 (see Table 3). Most (92%) of the included studies were more recent, published from 2019 and onwards [43–53].

**Table 3:** Descriptive results of studies.

Factor	Measure	Number of articles cited	Primary Author & Year
**Type of Study**			
	Cross-sectional	12	Bishwajit 2019; Gyasi 2020; Gogoi 2022; Koyanagi 2019; Pereira 2023; Rajkumar 2009; Selvamani 2023; Selvamani 2022; Smith 2022; Smith 2021a; Smith 2021b; T 2022
**Food Insecurity Outcome**			
	In the last 12 months, how often did you ever eat less than you felt you should because there wasn’t enough food?” and “In the last 12 months, were you ever hungry, but didn’t eat because you couldn’t afford enough food?”	6	Koyanagi 2019; Selvamani 2023; Smith, Shin 2021a; Smith 2021b; Smith 2022; Selvamani 2022
	Responses to 5-item questionnaire on food security adapted from Longitudinal Ageing Study in India survey	2	Gogoi 2022; Muhammad T. 2022
	“During the past 30 days, how often did you go hungry because there was not enough food in your home?”	1	Gyasi 2020
	Brazilian Food Insecurity Scale	1	Pereira 2023
	“In the last 12 months, how often did you ever eat less than you felt you should because there wasn’t enough food?”	1	Bishwajit 2019
	"experiencing hunger within the previous one month"	1	Rajkumar 2009
**Mental Health Outcome**			
	Depression	6	Pereira 2023; Rajkumar 2009; Smith 2021a; Smith 2021b; Bishwajit 2019; Muhammad T. 2022
	Psychological distress	2	Gogoi 2022; Gyasi 2020
	Suicidal Ideation	1	Smith 2022
	Perceived Stress	1	Selvamani 2022;
	Cognitive impairment	1	Koyanagi 2019
	Life Satisfaction	1	Selvamani 2022
**Age Studied**			
	50+	6	Bishwajit 2019; Gyasi 2020; Koyanagi 2019; Selvamani 2022; Smith 2022; Smith et al., 2021a
	60+	4	Gogoi 2022; Pereira 2023; Selvamani 2023; Muhammad T. 2022
	65+	2	Rajkumar 2009; Smith 2021b
**Study Findings**			
	Positive Association between mental health & food insecurity (for all levels of food insecurity)	8	Bishwajit 2019; Gyasi 2020; Gogoi 2022; Koyanagi 2019; Pereira 2023; Selvamani 2022; Selvamani 2023; Smith 2022
	Positive Association between mental health & food insecurity (did not specify severity)	2	Rajkumar 2009; Muhammad T. 2022
	Positive Association between mental health & food insecurity (Associated for severe FI only)	2	Smith 2021a; Smith 2021b
**Year published**			
	2023	2	Pereira 2023; Selvamani 2023
	2022	4	Gogoi 2022; Selvamani 2022; Smith 2022; Muhammad T. 2022
	2021	2	Smith 2021a; Smith 2021b
	2020	2	Bishwajit 2019; Gyasi 2020
	2019	1	Koyanagi 2019
	2009	1	Rajkumar 2009
**Location & World Bank Classification**			
	India (middle-income country)	8	Gogoi 2022; Rajkumar 2009; Selvamani 2023; Selvamani 2022; Smith 2022; Smith 2021a; Smith 2021b; Muhammad T. 2022
	South Africa (middle-income country)	7	Bishwajit 2019; Koyanagi 2019; Selvamani 2023; Selvamani 2022; Smith 2022; Smith 2021a; Smith 2021b
	Ghana (middle-income country)	6	Gyasi 2020; Selvamani 2023; Selvamani 2022; Smith 2022; Smith 2021a; Smith 2021b
	China (middle-income country)	5	Selvamani 2023; Selvamani 2022; Smith 2022; Smith 2021a; Smith 2021b
	Mexico (middle-income country)	5	Selvamani 2023; Selvamani 2022; Smith 2022; Smith 2021a; Smith 2021b
	Russia (middle-income country)	5	Selvamani 2023; Selvamani 2022; Smith 2022; Smith 2021a; Smith 2021b
	Brazil (middle-income country)	1	Pereira 2023

#### Spatial distribution of articles

**Fig 2:**
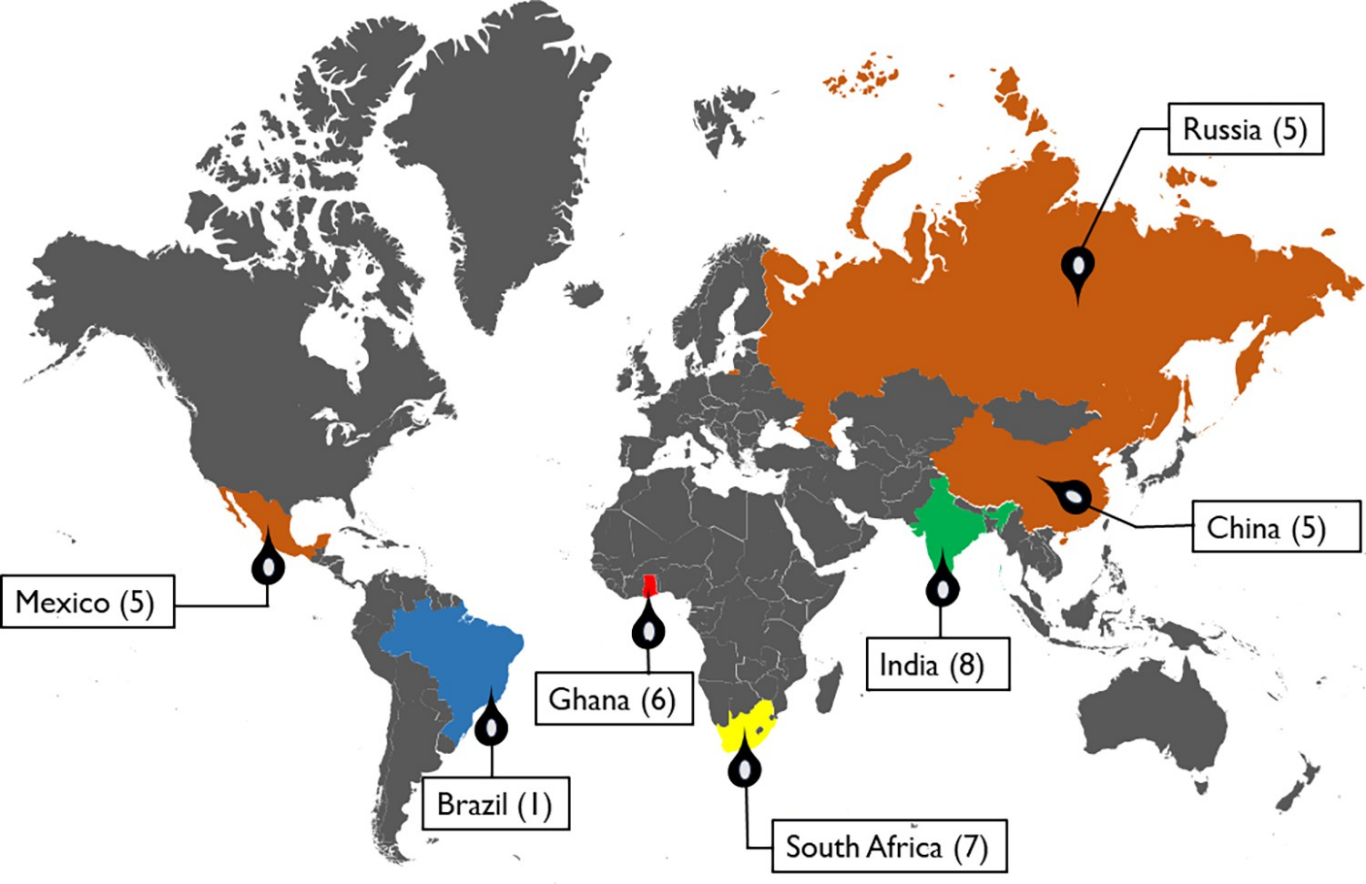
Spatial distribution of included studies. Basemap was adapted and modified from "Mapsvg". (Licensed under CC BY 4.0).

Furthermore, Fig 2 illustrates the spatial spread of articles included within this systematic review and the number of articles from each country. Five of the included studies used data from WHO’s Study on Global AGEing and Adult Health (SAGE), a longitudinal study collecting data on adults 50 and over from China, Ghana, India, Mexico, Russia, and South Africa [48–52]. In addition to these five studies, there were studies situated in India (3), South Africa (2), Ghana (1), and Brazil (1). India had the highest number of studies (61.5%) [45, 48–54]. All the countries are considered middle-income countries according to the World Bank Classification [55]. 10 of the 12 studies included in the review collected data from participants throughout the country, while the other two studies had a regional focus. For example, in one study, only older adults in the municipality of Barreiras in Bahia, Brazil were used [47]. Another study only focused on older adults from the Kaniyambadi block of Vellore district, a rural south Indian community [54].

#### Study design

All the studies were observational quantitative studies with a cross-sectional design (see Table 3).

#### Age and gender of sample

Among the studies, six considered older adults to be 50 and above [43, 44, 46, 49–51]. Four studies focused solely on adults aged 60 and [45, 47, 48, 53]. In contrast, the rest of the studies included in the review had participants aged 65 and above [52, 54]. All the studies involved male and female participants; however, only some of those studies (n = 3 differentiated the association between mental health and food insecurity by gender [43–45].

#### Outcome measures

A variety of mental health outcomes were measured in the included studies. The most common was depression (6), while other outcome measures included psychological distress (2), suicidal ideation (1), perceived stress (1), life satisfaction (1), and cognitive impairment (1) (see Table 3).

For food insecurity, the outcomes of the studies were related to hunger and food supply. Responses to the questions “In the last 12 months, how often did you ever eat less than you felt you should because there was not enough food?” and “In the last 12 months, were you ever hungry, but did not eat because you could not afford enough food?” were used to assess food insecurity for six of the included studies [46, 48–52]. One study used only the latter question for food insecurity [43]. Two studies used responses to a 5-item questionnaire on food security adapted from the Longitudinal Ageing Study in India survey as their food insecurity outcome [45, 53]. The study based in Brazil used the Brazilian Food Insecurity Scale [47]. Lastly, one study had participants answer, “experiencing hunger within the previous month?” [54].

### Major findings

The significant finding in all the included studies was that food insecurity is associated with the worsening mental health of older adults [43–54]. For ten of the studies [83%], the level of food insecurity was specified as severe or moderate [43–52]. For eight of the ten, moderate and severe food insecurity was significantly associated with various mental health outcomes [43–50]. Studies by [51, 52] concluded that moderate food insecurity was not significantly associated with worsening mental health but significantly with severe food insecurity. Two studies did not differentiate between the severity of food insecurity [53, 54]. In [53], the study had five different questions, each as an indicator of food insecurity. “Yes” to all the questions except “did not eat enough food of one’s choice” was significantly associated with major depression [53]. The authors found that those who answered yes to “reduced the size of meals” and “lost weight due to lack of food” had a stronger association with major depression compared to the other indicators of food insecurity [53]. The major themes that emerged from the analysis are shown in Table 4.

**Table 4:** Themes across included studies.

Theme	Number of articles cited	Primary Author & Year
Gender	3	Bishwajit 2019; Gyasi 2020; Gogoi 2022
Age	2	Gyasi 2020; Koyanagi 2019
Rural vs Urban	2	Gogoi 2022; Gyasi 2020
Health conditions	1	Selvamani 2022

### Major themes

#### Gender

The most common theme in the studies (n = 3, 25%) was comparing the association of food insecurity and mental health between males and females (Table 2). However, the results were inconsistent among them [43–45]. Two of the three studies indicated that the association between food insecurity and the mental health outcome studied was more robust for males than females [43, 44]. Gyasi et al. (2020), situated in Ghana, and Gogoi & Hazarika (2022), conducted in India, looked at psychological distress as the primary mental health outcome. Gyasi et al. [2020] found that for severe levels of hunger, the odds of psychological distress were higher among men compared to women. While in Gogoi & Hazarika (2022), the degree of the association between food insecurity and psychological distress was lower for men than women across different models adjusted for individual and household characteristics [45]. In Bishwajit et al. (2019), men in South Africa experiencing food insecurity had higher odds of depression than women [43].

#### Age

The second most common theme among the studies (n = 2, 15%) was age (Table 2). Gyasi et al. (2020) and Koyanagi et al. (2019) compared older adults aged 50–64 to older adults aged 65 and above [44, 46]. Both studies found that those who were 65 and above had worse mental health outcomes when experiencing severe food insecurity [44, 46]. In Gyasi et al. (2020), the association between severe food insecurity and psychological distress was more significant for those aged 65 years and above. Comparatively, in Koyanagi et al. (2019), for participants aged 65 years and above, severe food insecurity was associated with 3.87 times higher odds for mild cognitive impairment compared to 1.98 times higher odds with participants aged 50–64 [46].

#### Rural versus urban

One characteristic analyzed in Gogoi & Hazarika’s study (2022) was the participants’ location (rural vs urban. Participants for the study came from all states and territories in India except Sikkim. Of the 30,252 participants included in the study, 65.98% came from rural areas, compared to 34.02% from urban areas. The results showed that those who lived in rural areas and experienced moderate or severe food insecurity had higher levels of psychological distress than those living in urban places [45]. Additionally, in urban areas, psychological distress was only significantly associated with severe levels of food insecurity. Gyasi et al. (2020) also considered location’s impact on the association between food insecurity and mental health. The study was conducted in Ghana with 1200 participants. 55% of the participants came from urban areas, while 45% came from rural areas. Gyasi et al. (2020) did not find any significant difference in the association between food insecurity and mental health based on location of residence.

#### Health conditions

Given the prevalence of adverse health conditions among older adults, its mediation in the association between food insecurity and mental health is warranted. In the study by Selvamani & Arokiasamy (2022), the health conditions of participants were measured and analyzed. The health conditions involved self-reported health, self-reported difficulties in engaging in activities such as standing and climbing one flight of stairs, body mass index, lower back pain and chronic diseases [49]. Of the six countries involved in the study, three of them (India, Ghana, and South Africa) stated that there was an association between food insecurity and perceived stress that was significantly mediated by health conditions [49]. Specifically, for India, Ghana, and South Africa, self-reported health, self-reported difficulties in engaging in activities, body mass index, lower back pain, and chronic diseases mediated the association.

#### Fall-related injuries

A consequence of the association between food insecurity and mental health studied in one of the included studies is fall-related injuries [52]. The study included 14,585 participants aged 65 and above across six countries (China n = 5360, Ghana n = 1975, India n = 2441, Mexico n = 1375, Russia n = 1950, South Africa n = 1484) [52]. Participants who had suffered a fall as their most recent event of bodily injury in the past 12 months were considered to have had a fall-related injury [52]. In this study, severe food insecurity was significantly associated with 1.95 times higher odds of fall-related injuries [52]. Mental health was seen as a mediator between food insecurity and fall-related injuries. Mental health was broken down into anxiety, sleep problems, depression, and cognition. Through mediation analysis, anxiety, sleep problems, depression, and cognition represented 37.3%, 21.8%, 17.7%, and 14.0%, respectively, of the association between severe food insecurity and fall-related injuries [52].

## Discussion

This review examined the relationship between food insecurity and mental health among older adults residing in low- and middle-income countries. In all the included studies (12), food insecurity was significantly associated with poor mental health outcomes. The findings further reveal that a number of individual-level and socio-structural factors mediate and influence the deteriorative role of food insecurity on mental health.

### Broader associations

Overall, our findings are consistent with several other studies. In a systematic review studying the impact of food insecurity on mental health in Africa, it was found that there is a dose-response relationship between food insecurity and mental health [56]. There are several possible explanations for the impact of food insecurity on the mental health of older adults. In a study of chronic food insecurity in households in Burkina Faso, feelings of shame and guilt were consequences linked with food insecurity [57]. Shame can be experienced due to concern about one’s position in the social hierarchy or inability to feed oneself or family. Additionally, asking others for food is seen as socially unacceptable, which can increase feelings of shame, resignation and being overwhelmed [58]. These feelings can lead to symptoms of common mental disorders [59]. Food insecurity in older adults is also associated with loneliness and lack of social support [60–62]. Studies have shown that social support is protective against depression [63, 64]. Therefore, the lack of social support caused by social isolation in food-insecure individuals can lead to poorer mental health outcomes [60]. Another possible explanation for the association between food insecurity and mental health is the link between food and good memories. Food is associated with emotions, memories, and feelings of people, moments, and places from an older adult’s past experiences. Hence, when experiencing food insecurity, these individuals eat less food that typically gives them pleasure [47]. Food insecurity also leads to the disruptions of meal patterns, family rituals, and intra-generational transfer of knowledge and practices. These experiences often contribute to feelings of anxiety and worry that lead to poorer mental health outcomes [17]. Food insecurity is also associated with poor diet quality as individuals resort to cheaper, less unhealthy foods, which can increase the risk of mental health disorders, such as depression [46, 48, 65–67]. Poor diet has also been associated with an increased risk of cognitive decline, as saturated fat and sugar have been shown to trigger inflammation in the hippocampus, which can impair memory [46, 68]. Food insecurity can lead to a lower intake of fruits and vegetables, which contain essential nutrients, such as vitamins C, B, and E, that can protect against poor mental health outcomes [49, 67, 69]. Lastly, another possible explanation for the association between food insecurity and mental health is the impact of childhood malnutrition. Childhood food insufficiency significantly contributes to dementia in later years [69]. A study conducted on adolescents across 68 countries found that food insecurity can persist throughout life, as parents of food-insecure households were more likely to be food insecure during childhood [70]. Therefore, if food-insecure older adults were also food insecure during their childhood, this would contribute to poorer mental health outcomes in their older years.

### Gender

In this review, there were mixed findings on gender differences in the impact of food insecurity on the mental health of older adults. Two studies examining gender differences concluded that the association was stronger for men [43, 44]. While Gogoi & Hazarika (2022) found the degree of association between food insecurity and psychological distress was lower for men than women [45]. The ambiguity in this review are reflective of the existing literature. In a systematic review of the impact of food insecurity on mental health in Africa, five out of eight of the studies that looked at mixed-sex association found that the association was more significant for women [56]. In contrast, the other three studies concluded that there was no difference or more significant in men [56]. Furthermore, in a global analysis of 149 countries, it was found that sex did not modify the association between food insecurity and mental health [17]. A possible explanation for the mixed results throughout the literature is the differences in gender roles and cultural factors across the countries studied. Women in patriarchal societies commonly had less autonomy over resources and gender-based violence, which increased their experiences of food insecurity and impacted their mental health [56, 71–73]. Women were also primarily responsible for purchasing, cooking, and serving food to their families while eating last, and the inability to fulfill these tasks increased the risk of intimate partner violence [56, 74, 75]. This, in turn, could further impact their mental health [76–78]. The association between food insecurity and mental health can be more pronounced for men due to the role of men in some patriarchal societies. Men are seen as providers, so the inability to provide for their family resulting in food insecurity and having to ask for help, can lead to feelings of shame and inadequacy [56, 79, 80]. Men also often have less social support, which can increase the risk of food insecurity-related mental health issues [44, 56, 81].

### Age

The effect of age on the association between food insecurity and mental health was analyzed in two of the included studies [44, 46]. Both studies found that adults 65 and older had worse mental health outcomes while experiencing severe food insecurity than older adults aged 50–64 [44, 46]. Studies have shown that while there is an association between food insecurity and mental health for all age groups, there is a stronger association for older adults [17, 56]. Over the past few decades, there has been a decrease in familial support for older adults worldwide as older adults are less likely to live with adult children [56, 82]. Food insecurity has also been associated with functional disability among older adults [83–85]. As they age, decreases in mobility lead to less physical activity as well [86, 87]. The absence of these protective factors against poor mental health outcomes could explain why, as adults age, there is a more significant impact of food insecurity on their mental health [88–92]. As older adults age due to biological changes in their brains, such as smaller hippocampal volumes, they are at greater risk of cognitive declines that can be exacerbated by the consequences of food insecurity [46, 93, 94].

### Rural versus urban

One of the themes investigated in two of the included studies was the spatial location of participants, specifically whether there were rural-urban differences in the association between food insecurity and the mental health of older adults. One of the studies found a greater association between food insecurity and mental health for older adults who live in rural areas compared to older adults in urban areas [45]. The other study investigating this theme found no difference between urban and rural dwellers [44]. Research comparing the impact of food on mental health in urban and rural areas is limited. However, one study looking at the rural-urban differences in the association between food insecurity and cognitive impairment among older adults found that those experiencing food insecurity in rural areas had higher odds of cognitive impairment [95]. One reason the impact of food insecurity on the mental health of rural dwellers would be greater than that of urban dwellers is that rural dwellers are more likely to be farmers and produce their own foods [56]. If more rural dwellers are farmers and food insecurity hampers their ability to provide for their families financially, this could be a potential source of stress that is less likely to be experienced by urban dwellers [96, 97]. These additional financial stresses could lead to worsened mental health. Seasonality in relation to crops may be an additional factor that would impact the mental health of rural dwellers instead of urban dwellers. In Cole & Tembo (2011), it was suggested that the effect of food insecurity on mental health might be even greater during the rainy season if poor harvest causes negative feelings associated with social comparisons to food-secure neighbours and having to wait an additional year to increase resources [98].

### Health conditions

As health conditions vary amongst older adults, it is important to consider it when analyzing the association between food insecurity and mental health. In Selvamani & Arokiasamy (2022), the association between food insecurity and perceived stress was significantly mediated by health conditions such as self-reported health status, BMI, activities in daily life (ADL), low back pain and multimorbidity [49]. Lower back pain mediated the association between food insecurity and the mental health of older adults in India, Ghana, and South Africa [49]. Lower back pain is the most common pain experienced by older adults [99, 100]. Several studies have found that those with chronic back pain are more likely to be experiencing food insecurity [101, 102]. Qualitative studies found back pain to be associated with feelings of sadness and increasing experiences of social isolation [99, 100]. Chronic back pain is also significantly associated with higher odds of depression [50]. In Ziliak et al. (2008), it was stated that being marginally food insecure was roughly equivalent to being 14 years older in terms of limitations on activities in daily life [103, 104]. In Na & Streim (2017), there were significant decreases in most of the social network measures at the complete ADL limitations stage. As previously stated, decreased social support can lead to poor mental health outcomes. Higher states of ADL limitations were also associated with negative mental health outcomes [45, 105–107]. Several studies have reported an association between food insecurity and chronic diseases such as heart disease, diabetes, and hypertension [108–112]. The link between food insecurity and chronic diseases could be due to cyclically dysregulated eating patterns experienced during food insecurity characterized by overeating when food is available and reduced intake when food is unavailable; this could lead to metabolic disruption and then metabolic diseases [108, 112–114]. Diabetes is a common metabolic disease that can occur. It is suggested that peripheral insulin resistance, a precursor to diabetes, may emerge due to food insecurity [103, 110, 115]. Food insecurity is also often a stressful event that can raise cortisol levels [108–110]. Elevated cortisol levels are associated with visceral adiposity, which is a strong risk factor for diabetes [103, 108, 109]. Additionally, Seligman et al. (2012) found that food-insecure adults were more likely to report difficulties affording a diabetic diet than food-secure adults [103, 116]. This could lead to additional stress amongst food-insecure diabetics that could worsen mental health [116]. The association between diabetes and depression has been found in some studies [117–119]. In an epidemiological review of diabetes and depression, it was stated that people with diabetes are twice as likely to have depression than those without diabetes [119]. Furthermore, a systematic review of depression and type 2 diabetes in LMICs suggested that depression amongst people with diabetes is higher in those residing in LMICs compared to those in HICs [120].

### Fall-related injuries

The main theme in Smith et al. (2021b) was falls and how mental health complications mediate the association between food insecurity and fall-related injuries [52]. As seen throughout this review, food insecurity can negatively impact the mental health of older adults. Consequently, mental health issues can lead to a higher risk of experiencing falls [52, 121, 122]. Firstly, excessive fear of falling is often associated with depression, and it increases the odds of falling [122–124]. Mental health problems can lead to actual falls due to changes in gait [122, 125]. Generally, older adults need to pay more attention to walking to compensate for changes in their motor and sensory functions [122, 126]. Depression leads to changes in their ability to attend to their environment and is associated with increased unsteadiness [122, 127]. Medicines used to treat mental health issues, such as antidepressants, can cause adverse symptoms in older adults, which can lead to an increased chance of falling [122, 128, 129]. Research has shown that Vitamin D and calcium deficiencies and malnutrition are also risk factors for falls [52, 130–133]. This can also cause loss of muscle, strength, and the ability to do simple physical tasks due to less protein intake [52, 134, 135].

### Limitations

We have identified a few limitations of our systematic review. As both researchers are native English speakers, we only included studies in English, potentially missing out on studies not in English that could contribute to the systematic review. Due to the scarcity of research on certain themes we investigated in this review, some of the information used throughout the discussion was obtained from high-income countries like the U.S., which may not apply to LMICs as in lower-income countries, food insecurity may be associated more with hunger and undernutrition than obesity which could result in different effects on physical and mental health. All the studies included in this systematic review were cross-sectional studies [43–53].This is a limitation because the nature of cross-sectional studies does not allow us to make conclusions on causation, just whether there is a significant association between food insecurity and mental health. Furthermore, among the included studies, variations were observed in the time frames used to assess the prevalence of food insecurity (e.g., food security in the past month versus the past 12 months). These differences could potentially obscure the impact of food insecurity on mental health, as longer experiences of food insecurity might have a more significant effect on mental health. Many of the studies were also based on self-reported data, which could result in reporting or recall bias. Lastly, each study only underwent a quality assessment by one of the authors. Hence, it may have been more beneficial for both authors to assess the quality of the studies and compare assessments.

## Conclusion and policy recommendations

Despite the limitations outlined above, food insecurity has been increasing worldwide due to global events such as conflicts, where about 70% of those food insecure are in war-torn areas, and climate change [6]. Low- and middle-income countries are especially vulnerable to food insecurity. At the same time, the population of LMICs is ageing, as 67% of those aged 60 and above worldwide will reside in LMICs [34]. This highlights the need to investigate the impact of food insecurity on older adults in LMICs. Often, negative mental health outcomes as a consequence of food insecurity in LMICs are overlooked. This systematic review contributes to existing literature by investigating the impact food insecurity has on the mental health of older adults in LMICs.

To help older adults experiencing food insecurity and, consequently, poorer mental health conditions, we recommended some interventions. Firstly, governments in LMICs should consider providing unconditional cash transfers to older adults [136, 137]. Studies have shown unconditional cash transfers improve food security and mental health [138, 139]. Community gardens are another initiative that can be implemented to diversify diets further and increase the consumption of foods and vegetables [140, 141]. Increased training on agricultural activities, especially agroecological practices, could help improve nutrition awareness [142], healthy diets and kitchen gardening [143]. Another recommendation would be to implement home-delivery meal plans. In a systematic review by Gualtieri et al. (2018), home-delivering meal plans enhanced lower-class older adults’ diet and eating habits in parts of the USA [144]. Meal plans also help decrease stress [145] and anxiety linked to accessing food and decrease social isolation as older adults are able to communicate with meal-plan delivery volunteers [146].

### Suggestions for future research

After thoroughly investigating the literature, we have found some areas for future research. All seven countries included in our systematic review were middle-income countries. It is very important for more research on the effects of food insecurity on mental health to be investigated in low-income countries as food insecurity is more prevalent in low-income countries. Research conducted by the U.N. Food and Agriculture Organization found that 58% of people in low-income countries are food insecure [147]. Another area for future research is the need for more longitudinal studies to analyze the relationship between food insecurity and mental health in older adults over time. By conducting longitudinal studies, we can see which intervention best targets food insecurity and curbs negative mental health outcomes. The need for this research is especially evident for LMICs as most of the current research on the effects of government interventions on the association between food insecurity and mental health has been conducted in high-income countries. Food insecurity in older adults has also been shown to exacerbate medical conditions such as diabetes and poor health status, which are seen as risk factors for depression in older adults [53]. Therefore, an area of future research is to investigate the relationship between food insecurity and mental health for those with conditions prevalent in LMICs, such as diabetes and HIV/ AIDS. This is especially needed for LMICs as most of the research related to food insecurity and mental health in those with chronic diseases like diabetes is conducted in HICs, while 80% of people with type 2 diabetes worldwide reside in LMICs [120]. There is a gap in the current literature on the role mental health plays in the association between food insecurity and falls in LMICs. This is important as the risk of falls is a potential consequence of food insecurity that is more specific to older adults [148, 149]. Further, in recent years, the COVID-19 pandemic has caused increasing experiences of food insecurity and negative mental health outcomes worldwide [150, 151]. More recent research needs to be conducted on the effects COVID-19 had on the association between food insecurity and mental health on older adults in LMICs, especially since older adults were very vulnerable to the pandemic [152, 153]. Additional research, such as qualitative studies, must be conducted to understand the older adults’ perspectives better.

## Supporting information

S1 Checklist PRISMA 2020 checklist. (DOCX)
